# Performance of a Screening Mammography AI Algorithm Repurposed for Symptomatic Mammography in a Tertiary Outpatient Clinic

**DOI:** 10.3390/diagnostics16070984

**Published:** 2026-03-25

**Authors:** Helen Ngo, Eric Niller, Eric Schmitz, Elmar Kotter, Marisa Windfuhr-Blum, Claudia Neubauer, Ana-Luisa Palacios, Fabian Bamberg, Jakob Neubauer, Jakob Weiss, Caroline Wilpert

**Affiliations:** 1 Department of Diagnostic and Interventional Radiology, Medical Center—University of Freiburg, Faculty of Medicine—University of Freiburg, 79106 Freiburg, Germanyeric.schmitz@uniklinik-freiburg.de (E.S.);; 2 Lunit, 7F, 374, Gangnam-daero, Gangnam-gu, Seoul 06241, Republic of Korea

**Keywords:** artificial intelligence, mammography, breast cancer, symptomatic, diagnostic imaging, breast density, deep learning, computer-aided detection

## Abstract

**Background/Objectives**: The aim of the study was to evaluate the diagnostic accuracy of a commercial artificial intelligence (AI) algorithm originally developed for screening mammography when applied to symptomatic women presenting to a tertiary outpatient clinic. **Methods**: This single-center, retrospective diagnostic accuracy study included women who presented with breast symptoms to a tertiary outpatient clinic between January and June 2013 and underwent digital mammography. An AI algorithm cleared by the U.S. Food and Drug Administration (FDA)-cleared AI algorithm was applied to all mammograms and generated continuous malignancy scores ranging from 1 to 100. Mammographic breast density was classified according to the American College of Radiology Breast Imaging Reporting and Data System (BI-RADS) by two experienced radiologists. Histopathology, when available, or otherwise a minimum of 2 years of clinical and imaging follow-up served as the reference standard. Diagnostic performance was assessed using receiver operating characteristic (ROC) analysis with calculation of the area under the curve (AUC) and 95% confidence intervals (CI) derived by patient level bootstrap resampling (*n* = 2000). Analyses were performed for the overall cohort and stratified by breast density (non-dense [BI-RADS A–B] vs. dense [BI-RADS C–D]). **Results**: A total of 78 women (mean age, 55 ± 11 years) were included, of whom 16 had histopathological verification of suspicious lesions with proven breast cancer in 14 patients and 62 were classified based on follow-up alone. In the overall cohort (156 breasts, including 15 breasts with malignancies), the AI algorithm achieved an AUC of 0.96 (95% CI: 0.86–1.00). Performance remained high in non-dense breasts (AUC = 0.96; 95% CI: 0.88–1.00) and dense breasts (AUC = 0.99; 95% CI: 0.93–1.00), with no statistically significant difference observed between density subgroups (DeLong test, *p* = 0.36), although subgroup comparisons were underpowered. Decision curve analysis suggested a consistent positive net benefit across a wide range of threshold probabilities in both density groups. **Conclusions**: In this preliminary, single-center retrospective cohort, a screening-trained AI algorithm showed promising diagnostic accuracy when applied to symptomatic mammograms. These findings require validation in larger, contemporary, multicenter cohorts before clinical implementation.

## 1. Introduction

Breast cancer screening with mammography has been proven to reduce mortality by enabling detection of cancers at earlier, more treatable stages [1]. However, mammographic interpretation is challenged by non-negligible false-negative and false-positive rates, and radiologist performance shows substantial inter- and intra-reader variability [2]. Recent advances in artificial intelligence (AI) have led to the development of deep learning-based algorithms for mammographic cancer detection [3]. Standalone AI systems have demonstrated performance approaching or matching that of specialized radiologists in retrospective screening datasets. For example, an AI system evaluated on more than 2600 screening examinations achieved an area under the receiver operating characteristic curve (AUC) of 0.84, comparable to the average radiologist AUC of 0.81 in that study [4]. In a large multi-reader study, a commercially available AI algorithm showed no significant difference in cancer detection performance compared with 552 human readers (AUC approximately 0.93 vs. 0.88) [5].

AI has shown potential to improve screening efficiency by increasing specificity and reducing radiologist workload. In a recent population-based screening trial including approximately 89,000 women, an AI system-maintained sensitivity comparable to radiologists while substantially increasing specificity and lowering recall rates, resulting in a higher overall AUC (0.80 vs. 0.74) [6]. Another study reported that an AI model could outperform human readers in selected settings and reduce second-reader workload by up to 88% in a simulated double-reading workflow [3].

To date, however, most mammography AI algorithms have been developed and validated primarily in screening populations of asymptomatic women [7]. In routine clinical practice, mammography is also widely used in symptomatic women presenting with breast complaints such as palpable masses, pain, or nipple discharge. The diagnostic mammography setting differs substantially from screening, with a higher prevalence of cancer and a greater frequency of benign breast conditions such as cysts, fibroadenomas, or pronounced parenchymal density [8]. Symptomatic patients often undergo additional imaging and clinical correlation, and interpretation of mammograms may be influenced by knowledge of symptom location or clinical history. It therefore remains uncertain whether AI algorithms trained predominantly on screening examinations maintain their diagnostic performance when applied to this distinct clinical context. While cancers in symptomatic women may be larger or more conspicuous, potentially facilitating AI detection, the increased prevalence of benign abnormalities and atypical imaging patterns may conversely increase false-positive findings or obscure malignancies not conforming to typical screening presentations.

Given this gap in the evidence, we conducted a retrospective, exploratory study to assess the diagnostic performance of a screening-trained AI tool in a symptomatic patient population. We applied the Lunit INSIGHT MMG algorithm to standard mammograms of women presenting with breast symptoms at a tertiary outpatient breast clinic. Histopathology, when available, or a minimum of two years of clinical and imaging follow-up without evidence of malignancy served as the reference standard. We evaluated diagnostic performance using side-based receiver operating characteristic analysis, including assessment of the AUC in the overall cohort and stratification by breast density. In addition, we explored clinical utility using subgroup analyses to determine whether mammographic density influenced algorithm performance. We hypothesized that the AI algorithm would retain high discriminatory performance for malignancy (AUC > 0.9) in this symptomatic cohort, but that rigorous validation was necessary to identify potential limitations compared with its established screening performance. Our aim was to provide preliminary data to inform the potential clinical role of AI as a decision-support tool in diagnostic mammography and to identify safety- and equity-relevant considerations, including performance in dense breasts and in the context of symptomatic presentation.

## 2. Materials and Methods

### 2.1. Study Design and Population

This retrospective, single-center exploratory diagnostic accuracy study was conducted at an academic tertiary care outpatient breast clinic. The institutional ethics committee approved the study (approval number 22-1184), and the requirement for informed consent was waived.

We included all women who underwent bilateral two-view digital mammography (craniocaudal and mediolateral oblique views) between January and June 2013 for evaluation of breast symptoms. Breast symptoms were defined as the presence of at least one of the following: palpable abnormality, (asymmetric) mastodynia, or suspicious findings on prior sonographic examination.

Mammographic image quality was assessed by trained study staff under continuous supervision of a board-certified, subspecialty-trained breast radiologist with 13 years of experience in breast imaging. Clinical data were collected by specially trained study staff under the supervision of the same radiologist. Exclusion criteria were unavailable histopathologic verification and absence of clinical and imaging follow-up for at least two years, or severely impaired mammographic image quality precluding diagnostic interpretation.

### 2.2. Imaging and Breast Density Assessment

All mammograms were acquired using a full-field digital mammography system (Mammomat Inspiration; Siemens Healthineers, Erlangen, Germany). Breast density was assessed in consensus by two subspecialty-trained breast radiologists, each with more than 10 years of experience in breast imaging, according to the 5th edition of the American College of Radiology Breast Imaging Reporting and Data System (BI-RADS). For subgroup analyses, breast density was dichotomized into non-dense (BI-RADS A–B) and dense (BI-RADS C–D) categories. The breast radiologists, who were blinded to clinical data, also reviewed each mammogram and reached a consensus BI-RADS assessment (categories 1–5) reflecting the estimated likelihood of malignancy.

### 2.3. AI Algorithm and Output

The AI algorithm used was Lunit INSIGHT MMG v1.1.9.2 (Lunit Inc., Seoul, South Korea, Supplementary Figure S1), an FDA-cleared deep learning-based tool originally developed for screening mammography. The software generates continuous malignancy scores ranging from 1 to 100 for each breast. The AI tool was applied retrospectively to all included mammograms and was blinded to all clinical information, imaging reports, and reference standard outcomes.

### 2.4. Reference Standard

The reference standard was defined at the breast level. Histopathologic analysis of biopsies or surgical specimens served as the reference when available. In the absence of tissue sampling, a minimum of two years of clinical and imaging follow-up without evidence of malignancy was accepted as confirmation of benignity. Two experienced breast radiologists independently reviewed all imaging studies and corresponding medical records to assign reference outcomes, with discrepancies resolved by consensus.

### 2.5. Statistical Analysis

Continuous variables are presented as mean ± standard deviation, and categorical variables as frequencies and percentages. Diagnostic performance of the AI algorithm was assessed using receiver operating characteristic (ROC) analysis on a per-breast basis (i.e., treating the left and right breast of each woman as separate observations), with the area under the ROC curve (AUC) as the primary outcome measure.

AUCs and corresponding 95% confidence intervals (CIs) were estimated using patient-level bootstrap resampling (*n* = 2000) to account for within-patient correlation between breasts. ROC analyses were performed for the overall cohort and stratified by breast density (non-dense [BI-RADS A–B] vs. dense [BI-RADS C–D]). Differences in AUCs between density subgroups were assessed using the DeLong test. Clinical utility was further explored using decision curve analysis. As an additional sensitivity analysis, ROC/AUC estimates were recalculated in the subset of women with histopathologic verification, restricting the reference standard to tissue diagnosis.

All statistical analyses were performed using Python (version 3.11), with the scikit-learn and NumPy libraries. A two-sided *p*-value < 0.05 was considered statistically significant.

## 3. Results

### 3.1. Study Population

**Figure 1:**
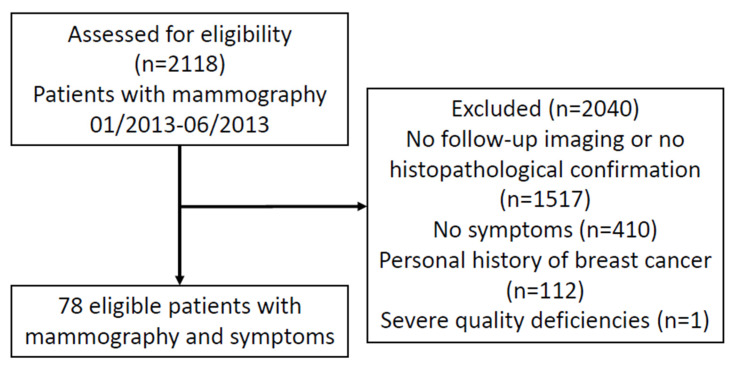
Flowchart of the study population selection and outcomes.

A total of 2118 women who underwent mammography at our tertiary center were assessed for eligibility. Of these, 1517 patients were excluded due to unavailable follow-up imaging or histopathological reports, 410 patients were excluded due to being without symptoms, and 112 patients were excluded due to a personal history of breast cancer. One additional patient was excluded because of severely impaired mammographic image quality. The final study cohort comprised 78 women (Figure 1). Although the included patients were significantly younger than the excluded patients, the absolute difference was modest, with mean ages of 55.5 ± 11.0 versus 58.9 ± 11.9 years, respectively (mean difference, −3.4 years; 95% CI, −5.9 to −0.9; Welch’s *t*-test, *p* = 0.0088; Mann–Whitney U test, *p* = 0.0083; Supplementary Table S1).

The mean age was 55.4 ± 11.0 years. Of the 78 included patients, 16 (20.5%) had histopathology available (with follow-up for the contralateral breast), while 62 (79.5%) patients were classified based on follow-up data only. As follow-up we only accepted our fully documented standardized assessment including physical breast examination, patient-reported symptom review, and bilateral imaging with mammography and breast ultrasound. This was independently reviewed by two breast radiologists who performed a consensus reading in cases of discrepancy.

### 3.2. Clinical and Imaging Characteristics

- The most common presenting symptom was mastodynia (*n* = 56, 71.8%), followed by palpable abnormalities (*n* = 14, 17.9%), and suspicious sonographic findings (*n* = 8, 10.3%).
- Menopausal status was premenopausal in 17 women (21.8%), postmenopausal in 35 (44.9%), perimenopausal in three (3.8%), and unknown in 23 (29.5%).
- Breast density was classified as BI-RADS A in 11 women (14.1%), B in 29 (37.2%), C in 33 (42.3%), and D in five (6.4%). Accordingly, 40 women (51.3%) had non-dense breasts (A–B) and 38 women (48.7%) had dense breasts (C–D).

### 3.3. Malignancy and Tumor Characteristics

- Histopathology confirmed malignancy in 14 of 78 patients (17.9%), while 64 patients (82.1%) had benign findings. Regarding each single breast, 15 of the 156 investigated breasts had malignant changes, including one case of bilateral disease.
- The most frequent histologic subtype was invasive carcinoma of no special type (NST) (*n* = 6, 42.9%), followed by invasive lobular carcinoma (ILC) (*n* = 4, 28.6%), ILC with lobular carcinoma in situ (LCIS) (*n* = 2, 14.3%), NST with associated ductal carcinoma in situ (DCIS) (*n* = 1, 7.1%), and pure DCIS (*n* = 1, 7.1%).
- Tumor staging revealed predominantly pT2 tumors (*n* = 7, 58.3%), followed by pT1 (*n* = 3, 25.0%) and pT4 (*n* = 2, 16.7%); no pT3 tumors were observed. Histologic grading showed a predominance of G2 tumors (*n* = 11, 78.6%), with one G1 (7.1%) and two G3 tumors (14.3%).
- Regarding receptor status, 12 tumors (85.7%) were estrogen- and progesterone-receptor positive. HER2 was positive in two tumors and negative in eleven; HER2 status was unavailable in one case. The Ki-67 proliferation index was >10% in 10 tumors (71.4%) and ≤10% in four tumors (28.6%) (Table 1).
- There were no high-risk lesions or triple negative cancers biopsied in this cohort.

### 3.4. Diagnostic Performance of the AI Algorithm

**Figure 2:**
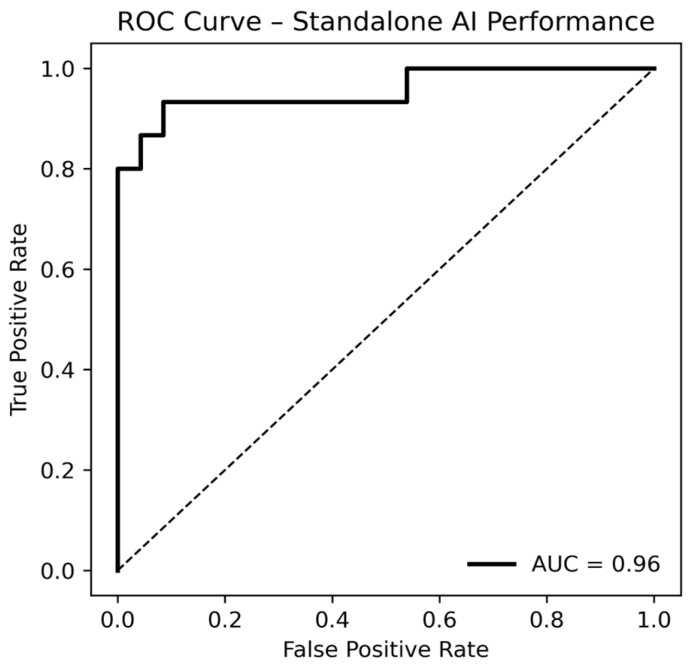
Receiver operating characteristic (ROC) curve illustrating the standalone performance of the AI algorithm (Lunit INSIGHT MMG) in symptomatic mammography. Side-based ROC analysis across the entire cohort yielded an area under the curve (AUC) of 0.96.

In the overall cohort, the AI algorithm achieved an AUC of 0.96 (95% CI: 0.86–1.00) across 156 breasts (15 with malignancies) (Figure 2).

**Figure 3:**
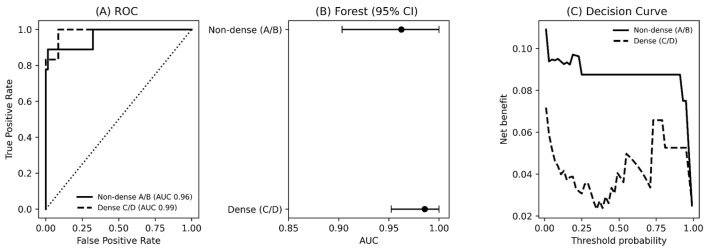
Diagnostic performance and clinical utility of the AI algorithm stratified by breast density. (**A**) Side-based receiver operating characteristic (ROC) curves for non-dense (BI-RADS A–B) and dense (BI-RADS C–D) breasts. (**B**) Forest plot showing areas under the curve (AUCs) with 95% confidence intervals. (**C**) Decision curve analysis suggesting net benefit across a range of threshold probabilities for both density subgroups. Higher net benefit indicates greater clinical utility (more true-positive cancers detected after accounting for the ‘cost’ of false-positive work-ups), and the threshold probability corresponds to the risk level at which a clinician would act.

When stratified by breast density, performance remained high in both subgroups. The AUC was 0.96 (CI 0.88–1.00) in non-dense breasts (BI-RADS A–B) and 0.99 (CI 0.93–1.00) in dense breasts (BI-RADS C–D). There was no statistically significant difference in diagnostic performance between density categories (DeLong test, *p* = 0.36). The exploratory decision curve analysis suggested a consistent positive net benefit across a wide range of threshold probabilities in both density subgroups (Figure 3). In Figure 3C, the threshold probability represents the minimum malignancy risk at which additional diagnostic work-up would be triggered, while the net benefit summarizes the trade-off between detecting cancers (true positives) and unnecessary work-ups (false positives), weighted by that threshold. The more pronounced U-shaped (and more jagged) curve in dense breasts indicates that clinical utility is more sensitive to the chosen threshold in this subgroup: at intermediate thresholds the balance between additional cancers detected and unnecessary work-ups is least favorable, whereas at higher thresholds net benefit increases again as the decision rule becomes more conservative. This non-monotonic pattern is expected with small subgroup sample sizes and should be interpreted cautiously.

In the histopathology-verified subset (17 breasts; 15 malignant breasts), the AI achieved an AUC of 0.90 (95% CI: 0.71–1.00) on a per-breast ROC analysis.

Using the pre-specified Lunit AI threshold (score ≥ 10 considered suspicious) and defining a positive human mammography assessment as BI-RADS 4–5, we performed a breast-level error analysis (156 breasts; 15 cancers). The AI produced one false-negative and 30 false-positive breasts (TP = 14, FN = 1, FP = 30, TN = 111), while human reading produced two false-negatives and one false-positive breast (TP = 13, FN = 2, FP = 1, TN = 140; Supplementary Figure S2). There was no overlap between AI and human error cases: 0 cancers were missed by both, and 0 benign breasts were flagged by both. Among the 15 cancers, 12/15 were detected by both AI and humans, 2/15 were detected only by AI, and 1/15 was detected only by humans.

Stratified by breast density (A/B = non-dense; C/D = dense), dense breasts comprised 76 breasts with six cancers (70 benign). In dense breasts, AI had 0/6 false-negatives and 26/70 false-positives, whereas humans had 1/6 false-negative and 0/70 false-positives. Non-dense breasts comprised 80 breasts with nine cancers (71 benign). In non-dense breasts, AI had 1/9 false-negative and 4/71 false-positives, while humans had 1/9 false-negative and 1/71 false-positive. Thus, AI false positives were concentrated in dense breasts (26/30, 87%), whereas human false positives were rare.

Detection also varied descriptively by presenting symptoms. For cancers in patients with a palpable abnormality, both AI and humans detected 10/10. In the absence of a palpable abnormality (five cancers), AI detected 4/5, while humans detected 3/5. For cancers in patients with a suspicious ultrasound at presentation (four cancers), AI detected 3/4 and humans detected 2/4; importantly, all cancers missed by either modality occurred in this “suspicious ultrasound” subgroup, whereas cancers without suspicious ultrasound were detected by both (11/11). Only one cancer occurred in a patient presenting with mastodynia, and it was detected by both.

With respect to mammographic lesion appearance, 13/15 (86.7%) cancers presented with a mass and 5/15 (33.3%) showed calcifications; calcifications always co-occurred with a mass (i.e., 0/15 calcification-only cancers). Specifically, 8/15 were mass-only, 5/15 were mass + calcifications, and 2/15 presented as asymmetry without mass or calcifications. For mass-associated cancers, AI detected 12/13, while humans detected 13/13; for cancers with calcifications (all mass-associated), both AI and humans detected 5/5. The two asymmetry-only cancers were detected by AI (2/2) but missed by humans (0/2), accounting for both human false-negative cancers in this dataset.

When combining density and lesion appearance, dense-breast cancers consisted of four mass-only, one mass + calcifications, and one asymmetry-only lesion: AI detected 6/6, while humans detected 5/6, missing the asymmetry-only lesion. Non-dense cancers consisted of four mass-only, four mass + calcifications, and one asymmetry-only lesion: AI detected 8/9, missing one mass-only cancer, while humans detected 8/9, missing the asymmetry-only cancer.

Regarding ultrasound visibility, all cancers were sonographically visible (15/15); therefore, no cancers were sonographically occult in this cohort. The three cancers missed by either modality (one AI false-negative and two human false-negatives) were all sonographically visible, including the single dense-breast cancer missed by human reading, indicating that ultrasound could plausibly have supported detection in these discordant/missed cases.

## 4. Discussion

In this small retrospective diagnostic accuracy study a screening-trained AI algorithm achieved an AUC of 0.96 (95% CI: 0.86–1.00) in identifying breast cancers in a cohort of symptomatic women. No statistically significant difference was observed between non-dense and dense breasts, although the subgroup analysis was underpowered. Although cross-study comparisons are limited by spectrum effects and case mix, and our findings must be interpreted cautiously given that the mammograms were acquired in 2013 and analyzed with a software version that may not reflect the current commercial release, the observed discrimination compares favorably with prior mammography AI reports, suggesting possible value of AI beyond its original intended use.

Our findings can be contextualized by comparing to prior studies of AI in breast imaging. In population screening settings, several studies have documented robust performance of AI algorithms. For instance, Rodriguez-Ruiz et al. showed that an AI system could match the average performance of 101 radiologists in screening detected cancers (AI AUC ~0.84) [4]. More recent large-scale evaluations have even suggested that AI may slightly outperform radiologist double reading in certain metrics: Chen et al. reported no difference in cancer detection AUC between an AI and hundreds of human readers, with the AI achieving higher specificity at a given sensitivity [5]. Furthermore, a study by Kwon et al. in a Korean screening cohort demonstrated that the Lunit AI had equivalent sensitivity to radiologists but dramatically higher specificity and a better overall AUC [6]. Against this backdrop, the AUC of 0.96 we observed in symptomatic women is notably high. High performance of AI in diagnostic/symptomatic environments has also been reported elsewhere: in symptomatic clinics in Hong Kong, an AI-CAD system achieved a median abnormality score of 95.6 with a sensitivity of 91.5% and specificity of 96.3% [9]. In a dedicated diagnostic mammography cohort, applying an optimized (higher) AI-CAD threshold yielded AUCs comparable to radiologists overall, and in symptomatic patients the AI-CAD AUC exceeded radiologists (0.873 vs. 0.815), with higher specificity/PPV at the optimized cutoff [10]. One reason could be the higher prevalence and generally larger size of malignancies in our symptomatic group (58% of cancers were T2 lesions), which might make them easier both for humans and AI to detect. It is worth noting that in an enriched dataset of challenging screening mammograms, an AI algorithm also achieved AUCs in the mid-0.90s [11], consistent with our result. Additionally, in a large multicenter cohort drawn mainly from clinics managing symptomatic breast patients, an AI system achieved AUCs ranging from 0.91 to 0.95 for distinguishing malignant from non-malignant breasts, supporting strong performance in diagnostic settings [12]. Overall, the high AUC observed in this cohort is consistent with reports of strong AI performance in diagnostic settings. These findings are preliminary and require validation in larger, contemporary, multicenter cohorts before generalizing performance to broader symptomatic populations.

Another interesting point from our study is that the AI’s accuracy did not appear to degrade in dense breasts, even though dense tissue is traditionally associated with reduced sensitivity for human readers. Dense breast tissue has been known to obscure cancers on mammography and has prompted legislative actions for supplemental screening in many regions [13]. AI tools could potentially help mitigate this disparity if they enhance performance in dense breasts. In a large screening study, Lunit’s algorithm showed a consistently higher specificity than radiologists in all density groups, including heterogeneously dense and extremely dense breasts [6]. Other work has similarly noted that while AI and radiologists both see some drop in sensitivity with increasing density, AI’s overall accuracy and its ability to reduce false positives remain beneficial even in dense breasts [11]. Our results add to this evidence, suggesting that at least for the types of cancers present in our symptomatic sample, parenchymal density did not appear to be a major obstacle for the AI. However, symptomatic-clinic data have reported that false-negative AI-CAD cases were more common in dense breasts and often presented as asymmetries, highlighting that density-related masking may still matter depending on lesion phenotype and case mix [9]. We cannot definitively conclude that the algorithm would perform equally well in a larger set of extremely dense breasts or for detecting the most occult tumors masked by dense tissue. Caution is warranted in generalizing these density observations, and further studies should specifically examine AI performance in dense breast symptomatic populations. Nonetheless, the lack of a drop off in our study is encouraging, as it hints that the algorithm may provide more uniform diagnostic support across different patient subgroups, an important consideration for health equity in AI deployment.

The clinical implications of using AI in a breast clinic for symptomatic patients are numerous. Our institution’s standard practice for diagnostic mammography is double reading by two radiologists, given the higher stakes and often complex cases that present with symptoms. Incorporating AI as an adjunct or triage tool could potentially streamline this workflow. For example, AI might serve effectively as a prereader or concurrent “second reader”, flagging suspicious findings that a single human reader should not miss, or alternatively, rapidly clearing obviously normal exams to focus radiologist attention where it is needed. With appropriate threshold tuning, it might even be used to triage symptom driven imaging: for example, an AI could identify the subset of mammograms with very low suspicion (allowing quicker discharge or focus on other modalities) while ensuring that any case with even moderate suspicion is reviewed thoroughly by radiologists and supplemented with ultrasound or MRI. This is consistent with diagnostic data showing that a screening-oriented vendor threshold can be suboptimal, whereas a higher, ROC-optimized threshold improves specificity, accuracy and PPV without compromising sensitivity—especially in symptomatic indications [10]. This concept is analogous to proposed AI triage in screening programs, where low risk exams may not require double reading [3]. Prospective population-based evidence shows that substituting one human reader with AI can be non-inferior for cancer detection while enabling controlled implementation pathways [14]. Any such operational use would require prospective validation, but our data provide an initial signal that the tool is both sensitive and highly specific in a scenario where pretest probability of disease is much higher than in screening.

It is important to discuss the limitations and safety considerations of using AI in symptomatic populations. First, unlike radiologists, the AI does not incorporate clinical history or symptom information when interpreting images; it analyzes the pixels in isolation. Symptomatic assessment often benefits from knowing where a patient feels a lump or pain. Radiologists correlate the physical exam or symptom location with imaging findings. An AI system, by design, lacks this context. A recent study highlighted that human readers significantly improve mammography interpretation accuracy when clinical symptoms are available, whereas an AI operating alone cannot leverage that information [15]. In practical terms, this means that if a patient reports a palpable mass but the AI score is low, a radiologist should not simply dismiss the concern based on the AI. Careful clinical correlation and additional imaging (targeted ultrasound, etc.) remain essential. Indeed, Choi et al. observed that certain cancers in symptomatic women were detected only by radiologists who were aware of the patient’s symptoms, whereas the AI tool missed them due to subtle imaging signs [15]. This underscores an ethical point: AI used off-label in diagnostic settings must be integrated judiciously, with the understanding that it is an aid and not a replacement for comprehensive clinical evaluation. We strongly caution against an overreliance on AI in symptomatic cases. If the AI output is negative but clinical suspicion is high, standard care dictates that patients still undergo further appropriate diagnostic work-up. Ensuring that the deployment of AI does not inadvertently delay diagnoses in symptomatic women (for example, by incorrectly reassuring providers or patients) is a paramount concern.

Our study has several limitations that temper the interpretation of the results. Accordingly, these findings should be interpreted as preliminary/hypothesis-generating. This was a single-center, retrospective study with a modest sample size (78 women, 14 cancers). The small sample—together with the tertiary referral setting and the high exclusion rate due to missing follow-up/histopathology—limits generalizability and may introduce selection bias toward patients with more complete documentation and standardized work-up. The data were from a tertiary referral clinic and from the year 2013; thus, the patient population may not perfectly reflect current practice or broader community settings. AUC estimates remained high in the histopathology-only sensitivity analysis, consistent with the primary findings. However, precision is limited by the small number of histopathology-confirmed cases. The AI algorithm we used (Lunit INSIGHT MMG) will likely undergo updates from the version applied to our retrospective dataset, and performance could differ with newer versions or on more diverse data. In routine diagnostic work-up of symptomatic women, radiologists interpret mammograms in the context of symptom location, physical examination findings, prior imaging, and correlative modality (most commonly targeted ultrasound and, when indicated, MRI) information that is not available to a standalone AI model [15]. In particular, the evaluated AI output did not incorporate comparison with prior mammograms and therefore could not assess interval change, a key component of diagnostic mammography interpretation. The direct head-to-head comparison to routine care would be confounded by access to multimodal and clinical information unavailable to standalone AI. Also, forcing radiologists to read mammograms in isolation does not reflect real-world diagnostic pathways. However, our evaluation focused on mammography-only standalone performance; we did not assess multimodal integration of AI outputs with ultrasound or MRI, nor whether AI might reduce downstream imaging in low-risk symptomatic presentations. Future research should explore the algorithm’s role in the full diagnostic pathway, perhaps as a decision-support tool for whether additional imaging is warranted. We also note that using an AI in a population different from the one it was originally validated on is effectively an off-label-use. Ethically, deploying a screening-trained tool for symptomatic indications raises concerns about automation bias, accountability, and equity. We did not have sufficient power to assess performance differences across important subgroups beyond density, and we did not evaluate calibration or downstream clinical consequences of acting on AI scores. Implementation would therefore require clear communication of intended use, human oversight, and ongoing monitoring with software version changes.

## 5. Conclusions

In this small, retrospective single-center cohort, a screening-trained AI algorithm achieved an AUC of 0.96 when applied to symptomatic mammograms, with high specificity and good sensitivity. These findings suggest promising diagnostic accuracy, but require validation in larger, contemporary, multicenter cohorts before generalizing performance or clinical utility. These results suggest the possibility of AI to become an adjunct in diagnostic breast imaging, with possible benefits including workload reduction and consistent performance across varying breast densities. However, the retrospective nature and limited scope of our study mean that these findings should be confirmed in larger, multicenter cohorts. Moreover, integration of AI into symptomatic workflows must be done carefully, respecting the nuances of clinical context and ensuring that the technology is used to support, rather than supplant, the comprehensive care that symptomatic patients require. With further validation, AI may become an important tool in the radiologist’s armamentarium for complex breast clinics, improving efficiency and possibly diagnostic confidence, while upholding the standard of care through mindful application and oversight.

## Figures and Tables

**Table 1:** Clinicopathologic characteristics and reference-standard outcomes of the study cohort.

Variable	Category	*n*/N (%)
Reference standard	Histopathology available	16/78 (20.5%)
	Follow-up only	62/78 (79.5%)
Outcome (patients)	Malignant	14/78 (17.9%)
	Benign	64/78 (82.1%)
Outcome (breasts)	Malignant breasts (side-based)	15/156 (9.6%)
	Benign breasts (side-based)	141/156 (90.4%)
Laterality among cancers (*n* = 14)	Right only	5/14 (35.7%)
	Left only	8/14 (57.1%)
	Bilateral	1/14 (7.1%)
Histology among cancers (*n* = 14)	Invasive carcinoma NST	6/14 (42.9%)
	Invasive lobular carcinoma (ILC)	4/14 (28.6%)
	NST with DCIS	1/14 (7.1%)
	ILC with LCIS	2/14 (14.3%)
	DCIS	1/14 (7.1%)
T stage among cancers (*n* = 14)	T1	3/14 (21.4%)
	T2	7/14 (50.0%)
	T3	0/14 (0.0%)
	T4	2/14 (14.3%)
	Missing	2/14 (14.3%)
Grade among cancers (*n* = 14)	G1	1/14 (7.1%)
	G2	11/14 (78.6%)
	G3	2/14 (14.3%)
Receptor status (*n* = 14)	ER positive	12/14 (85.7%)
	PR positive	13/14 (92.9%)
	ER+/PR+	12/14 (85.7%)
HER2 (*n* = 14)	Positive	2/14 (14.3%)
	Negative	11/14 (78.6%)
	Missing	1/14 (7.1%)
Ki-67 (*n* = 14)	>10%	10/14 (71.4%)
	≤10%	4/14 (28.6%)

## Data Availability

The data presented in this study are available on reasonable request from the corresponding author due to ethical and privacy restrictions.

## Supplementary Materials

The following supporting information can be downloaded at: https://www.mdpi.com/article/10.3390/diagnostics16070984/s1, Supplementary Table S1, Supplementary Figures S1 and S2. Supplementary Table S1: Baseline Demographic Characteristics of the Included and Excluded Patient Cohorts. Supplementary Figure S1: Inference workflow of the Lunit INSIGHT MMG mammography AI. Bilateral craniocaudal (CC) and mediolateral oblique (MLO) mammograms are exported in DICOM format, de-identified and preprocessed, and analyzed by a deep learning convolutional neural network. The algorithm outputs heatmap/marks highlighting suspicious regions and lesion-level scores (1–100). A per-breast abnormality score (1–100) is reported as the maximum lesion score across the CC and MLO views for each breast, and results are stored as DICOM overlays for review after the radiologist’s initial read. Supplementary Figure S2: Discordant and concordant case examples comparing an AI risk score with radiologist BI-RADS assessment on full field digital mammography. (A) Discordant interpretation. Mammography of a woman in her 80s presenting with a palpable right breast mass. Clinical examination showed right nipple retraction and a large upper outer quadrant mass adherent to the pectoralis. Histopathology confirmed a large breast carcinoma infiltrating the chest wall; the lesion is only partially captured on the mammographic views. The AI system assigned low/benign scores to both breasts (no suspicious finding), whereas radiologists assessed the right breast as BI-RADS 5. Bilateral plasma cell mastitis is visible; an enlarged left lymph node led to a BI-RADS 3 assessment on the left (benign on further work-up). (B) Concordant interpretation. Mammography of a woman in her 40s with a palpable left breast mass. The AI system assigned high malignancy scores to the left breast (98–99) with an unremarkable score on the right, matching radiologists’ assessments (left BI-RADS 5; right BI-RADS 1). Histopathology confirmed an invasive breast carcinoma on the left. CC, craniocaudal; MLO, mediolateral oblique; BI-RADS, Breast Imaging Reporting and Data System.

## Abbreviations

The following abbreviations are used in this manuscript:

AI	Artificial Intelligence
AUC	Area Under the Curve
BI-RADS	Breast Imaging Reporting and Data System
CAD	Computer-Aided Detection
CI	Confidence Interval
DCIS	Ductal Carcinoma in Situ
ER	Estrogen Receptor
FDA	Food and Drug Administration
HER2	Human Epidermal Growth Factor Receptor 2
ILC	Invasive Lobular Carcinoma
LCIS	Lobular Carcinoma In Situ
MRI	Magnetic Resonance Imaging
MMG	Mammography
NST	No Special Type
PR	Progesterone Receptor
ROC	Receiver Operating Characteristic
SD	Standard Deviation
