# Supplementary material for: Performance of a Screening Mammography AI Algorithm Repurposed for Symptomatic Mammography in a Tertiary Outpatient Clinic
Source: Diagnostics (Basel). 2026 Mar 25;16(7):984. doi: 10.3390/diagnostics16070984 (PMC13072339; doi:10.3390/diagnostics16070984)
Supplement: Supplementary file 1 [file diagnostics-16-00984-s001.zip › Supplementary_Figures 1 and 2.pdf]

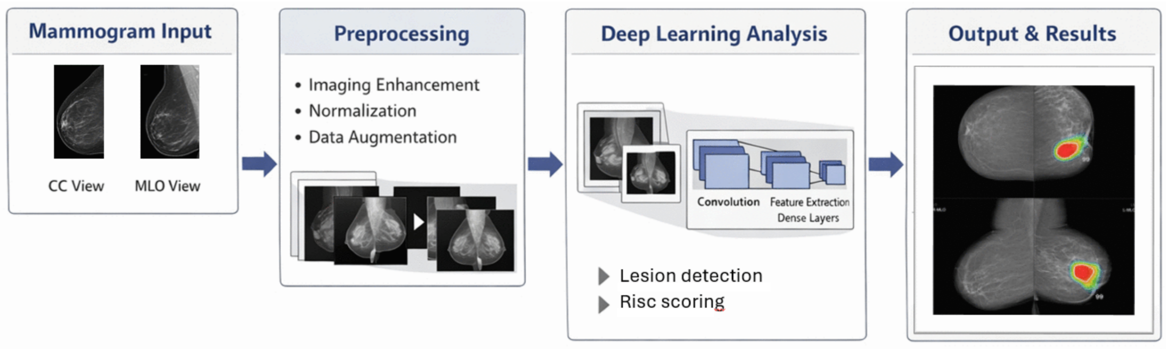

Supplementary Figure S1. Inference workflow of the Lunit INSIGHT MMG mammography AI. Bilateral craniocaudal (CC) and mediolateral oblique (MLO) mammograms are exported in DICOM format, de-identified and preprocessed, and analyzed by a deep learning convolutional neural network. The algorithm outputs heatmap/marks highlighting suspicious regions and lesion-level scores (1–100). A per-breast abnormality score (1–100) is reported as the maximum lesion score across the CC and MLO views for each breast, and results are stored as DICOM overlays for review after the radiologist's initial read.

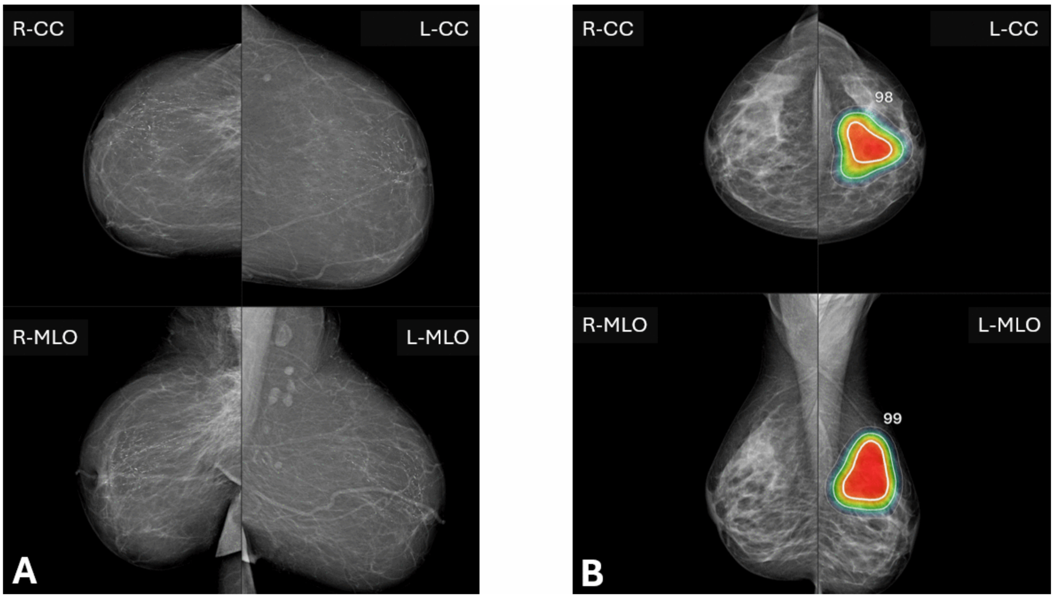

Supplementary Figure S2. Discordant and concordant case examples comparing an AI risk score with radiologist BI-RADS assessment on full-field digital mammography.
(A) Discordant interpretation. Mammography of a woman in her 80s presenting with a palpable right breast mass. Clinical examination showed right nipple retraction and a large upper-outer quadrant mass adherent to the pectoralis. Histopathology confirmed a large breast carcinoma infiltrating the chest wall; the lesion is only partially captured on the mammographic views. The AI system assigned low/benign scores to both breasts (no suspicious finding), whereas radiologists assessed the right breast as BI-RADS 5. Bilateral plasma cell mastitis is visible; an enlarged left lymph node led to a BI-RADS 3 assessment on the left (benign on further work-up).
(B) Concordant interpretation. Mammography of a woman in her 40s with a palpable left breast mass. The AI system assigned high malignancy scores to the left breast (98–99) with an unremarkable score on the right, matching radiologists' assessments (left BI-RADS 5; right BI-RADS 1). Histopathology confirmed an invasive breast carcinoma on the left.

CC, craniocaudal; MLO, mediolateral oblique; BI-RADS, Breast Imaging Reporting and Data System.
