# Supplementary material for: Performance of a Screening Mammography AI Algorithm Repurposed for Symptomatic Mammography in a Tertiary Outpatient Clinic
Source: Diagnostics (Basel). 2026 Mar 25;16(7):984. doi: 10.3390/diagnostics16070984 (PMC13072339; doi:10.3390/diagnostics16070984)
Supplement: Supplementary file 1 [file diagnostics-16-00984-s001.zip › Suppl_Table_S1.pdf]

Supplementary Table S1. Baseline Demographic Characteristics of the Included and Excluded Patient Cohorts.

Feature	Included	Excluded
Unique patients	78	2040
Age, mean ± SD	55.5 ± 11.0 years	58.9 ± 11.9 years
Age, median [IQR]	55.2 [47.0; 62.5] years	58.8 [50.4; 68.2] years
Age, range	27.6–89.7 years	23.4–90.4 years
